# Early cost-effectiveness of tumor infiltrating lymphocytes (TIL) for second line treatment in advanced melanoma: a model-based economic evaluation

**DOI:** 10.1186/s12885-018-4788-5

**Published:** 2018-09-15

**Authors:** Valesca P. Retèl, Lotte M. G. Steuten, Marnix H. Geukes Foppen, Janne C. Mewes, Melanie A. Lindenberg, John B. A. G. Haanen, Wim H. van Harten

**Affiliations:** 1 Division of Psychosocial Research and Epidemiology, Netherlands Cancer Institute-Antoni van Leeuwenhoek Hospital (NKI-AVL), Plesmanlaan 121, 1066 CX Amsterdam, the Netherlands; 2 Department of Health Technology and Services Research, University of Twente, Postbus 217, 7500 AE Enschede, the Netherlands; 3 Fred Hutchinson Cancer Research Center, 1100 Fairview Avenue North, P.O. Box 19024, Seattle, WA 98109-1024 USA; 4 Department of Medical Oncology, Netherlands Cancer Institute-Antoni van Leeuwenhoek Hospital (NKI-AVL), Plesmanlaan 121, 1066 CX Amsterdam, the Netherlands

**Keywords:** Tumor infiltrating lymphocytes, Ipilimumab, Advanced melanoma, Cost-effectiveness analysis, Coverage with evidence development, Personalized medicine

## Abstract

**Background:**

An emerging immunotherapy is infusion of tumor infiltrating Lymphocytes (TIL), with objective response rates of around 50% versus 19% for ipilimumab. As an Advanced Therapeutic Medicinal Products (ATMP), TIL is highly personalized and complex therapy. It requests substantial upfront investments from the hospital in: expensive lab-equipment, staff expertise and training, as well as extremely tight hospital logistics. Therefore, an early health economic modelling study, as part of a Coverage with Evidence Development (CED) program, was performed.

**Methods:**

We used a Markov decision model to estimate the expected costs and outcomes (quality-adjusted life years; QALYs) for TIL versus ipilimumab for second line treatment in metastatic melanoma patients from a Dutch health care perspective over a life long time horizon. Three mutually exclusive health states (stable disease (responders)), progressive disease and death) were modelled. To inform further research prioritization, Value of Information (VOI) analysis was performed.

**Results:**

TIL is expected to generate more QALYs compared to ipilimumab (0.45 versus 0.38 respectively) at lower incremental cost (presently €81,140 versus €94,705 respectively) resulting in a dominant ICER (less costly and more effective). Based on current information TIL is dominating ipilimumab and has a probability of 86% for being cost effective at a cost/QALY threshold of €80,000. The Expected Value of Perfect Information (EVPI) amounted to €3 M.

**Conclusions:**

TIL is expected to have the highest probability of being cost-effective in second line treatment for advanced melanoma compared to ipilimumab. To reduce decision uncertainty, a clinical trial investigating e.g. costs and survival seems most valuable. This is currently being undertaken as part of a CED program in the Netherlands Cancer Institute, Amsterdam, the Netherlands, in collaboration with Denmark.

## Background

Until recently metastatic melanoma was almost uniformly fatal, with a median survival of 9 months [1]. In 2011 ipilimumab, a monoclonal antibody against CTLA-4 on the activated T-lymphocyte was the first newly-introduced treatment that increased survival in this group of patients. Very recently anti-programmed cell death-1 (PD-1) antibodies, such as nivolumab and pembrolizumab, were introduced as first line treatment, as these led to an even longer progression free and overall survival [2–5]. Therefore, ipilimumab is now most often used as second line treatment. On average, ipilimumab extends survival by 3.6 months (when compared to a gp100 peptide vaccine), and increases the 1-year survival rate from previously 25.5% to 44% of patients [6]. However, the treatment costs are high, around €80,000 per patient, and 10–20% of patients treated with ipilimumab have serious immune-related adverse events.

Adoptive T-cell therapy (ACT), in particular the ACT variant Tumor Infiltrating Lymphocytes (TIL) is a powerful immunotherapy directed against metastatic melanoma. A number of nonrandomized clinical trials testing TIL have consistently found clinical response rates of around 50% in metastatic melanoma patients, accompanied by long progression-free survival (PFS). Indeed, complete remission is achieved in 10–20% of patients treated with TIL. The overall response rates range from 35 to 72%, with more than 20% of the treated patients surviving more than 3 years [7]. Andersen et al. reported 1- and 3-year survival rates of 72% and 40.8% respectively [8]. Other studies have also established practical methods for the expansion (growth) of TILs from melanoma tumors with high success rates [9–11]. Side effects of TIL as observed in these trials were manageable, and the costs for the treatment are around €60,000 per patient.

Notwithstanding the expectation that TIL effectively outperforms ipilimumab, it is a complex process. Stringent eligibility criteria apply for TIL, such as having a resectable tumor, adequate heart and lung function and no or very limited and asymptomatic brain metastases. This means that TIL can be used for approximately 50% of advanced melanoma patients. On the resources side, as known for Advanced Therapeutic Medicinal Products (ATMPs), TIL requests substantial upfront investments from the hospital in e.g. a specialized GMP laboratory necessary to culture the TILs, expensive lab-equipment, trained and experienced technical staff, as well as extremely tight hospital logistics [12]. Because a cell product is being made specifically for every individual patient, such treatments may not be commercially interesting for the pharmaceutical companies to explore. The total trajectory of one TIL-treatment may take as long as 3 weeks of highly personalized, labour-intense treatment, including side effect management, which has to be provided partly in a specialized inpatient setting. Therefore, TIL was first implemented in specialized cancer centres like the National Cancer Institute (Bethesda, USA) and some others worldwide, including the Netherlands Cancer Institute (NKI). So far, the investments for the necessary infrastructure were made in the (non-profit) hospital and are therefore hard to recoup. A Coverage with Evidence Development (CED) program for TIL as second line treatment for advanced melanoma was started in 2015 and lead by the NKI. CED provides payment for new and/or innovative treatments while simultaneously generating clinical data to demonstrate the treatment’s effect on health outcomes, including early stage economic evaluation [13]. The goal of the program is to support innovation and the timely collection of data while helping payers to take evidence-based decisions that improve health outcomes.

Considering that TIL (in phase II trials) has longer overall and progression-free survival compared to ipilimumab against lower treatment costs, we hypothesize that TIL is more cost-effective than ipilimumab. Since these treatments have not yet been compared head-to-head, the aim of this study is to estimate the cost-effectiveness of TIL versus ipilimumab in second line of treatment, as well as to analyse the value of further research.

## Methods

The aim of this study is to estimate the (early) cost-effectiveness of TIL versus ipilimumab for second line of treatment in advanced melanoma patients, as well as to analyse the value of further research. The design is a model-based Cost-Effectiveness Analysis, in the setting of the Netherlands.

### Model description

A Markov model was developed with three health states to compare TIL versus ipilimumab. A hypothetical cohort of 1000 patients with metastatic melanoma (stage IV) was simulated, starting at age 52 (average age at diagnosis measured in a pilot study in the Netherlands Cancer Institute) in the health state “stable disease”. From there, patients can go to the health states “progressive disease”, and the absorbing state “death”. Patients in “progressive disease” either stay there or die. “Progressive disease” is defined according to the Response Evaluation Criteria In Solid Tumors (RECIST) 1.1 criteria [14]. The analysis was conducted from the Dutch health care system perspective and has a 10-year time horizon, with 10 cycles of 1 year. The primary outcome measures of the analysis were costs per life-year gained and costs per quality adjusted life years (QALY) gained. The analysis was performed according to the Consolidated Health Economic Evaluation Reporting Standards (CHEERS) guidelines for cost-effectiveness analysis [15]. Because this study is based on literature, Institutional Review Board was not necessary.

### Treatments compared

**Fig. 1:**
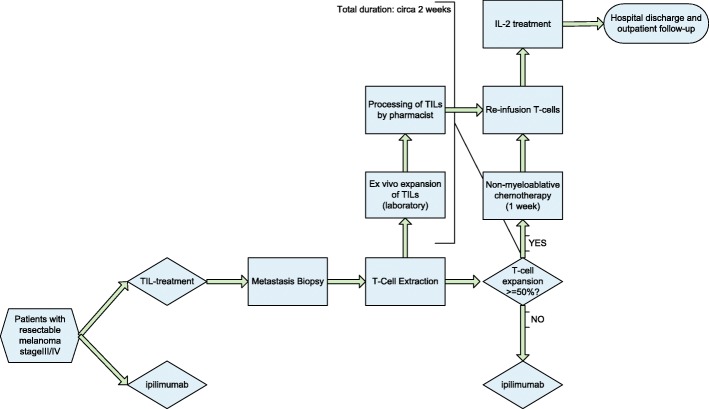
Flowchart TIL treatment

In the TIL-group, according to the protocol, patients first undergo surgical resection of a metastasis. From this resection, TILs are grown in vitro, and then expanded. Before re-infusion of the grown TILs, patients undergo non-myeloablative lymphocyte depleting chemotherapy consisting of cyclophosphamide (60 mg/kg/day × 2 days i.v.) and fludarabine (25 mg/m^2^/day IV × 5 days). The TILs are reinfused to the patient, followed by high-dose bolus interleukin-2 (600.000 IU/kg/dose every 8 h, up to 15 doses). The patients receive blood or platelet transfusion until spontaneous hematopoietic recovery occurs. Pegfilgrastim injections are initiated the day after TIL infusion until neutrophil recovery. In the ipilimumab group, patients receive ipilimumab, 3 mg/kg, every 3 weeks, with a maximum of four cycles (Fig. 1). The recommended dose of ipilimumab is 3 mg/kg bodyweight administered intravenously over a 90-min period every 3 weeks. Four cycles form a full treatment course [16].

### Transition probabilities

Transition probabilities were calculated from the data of published studies. Data on the effectiveness of TIL was obtained from two phase II studies [10, 11]. These studies were the only two which reported progression free survival (PFS) and overall survival (OS), and resemble mostly the current given TIL treatment. From these studies (*n* = 20 [10] and *n* = 31 [11]) the data was pooled. CMA Software, version 3, Biostat, USA, was used to calculate the 95%b Confidence Intervals (95% CIs) of the data [17]. The 1y overall survival (OS) rate measured by Besser et al. was 0.350 (7 out of 20, (95% CI 0.177–0.574), and 0.645 (20 out of 31, CI 0.466–0.791) measured by Radvanyi et al. The progression free survival (PFS) rate was 0.150 (3 out of 20, CI 0.049–0.376), and 0.323 (10 out of 31, CI 0.183–0.503) respectively. The pooled analysis resulted in an OS rate of 0.531 (CI 0.389–0.667) and a PFS rate of 0.266 (CI 0.160–0.408). Data on the effectiveness of ipilimumab were based on a randomised controlled trial [6], in which the OS and PFS of patients randomized into either ipilimumab plus gp100, ipilimumab alone, or gp100 alone was evaluated. For our analysis, we used the 1-year OS rate for ipilimumab alone: 0.456 (CI 0.218–0.311) and for the 1-year PFS rate: 0.192 (CI 0.163–0.298). (Table 1) Besides the difference of the level of evidence (phases II and III), the patient population included in all three studies were comparable as they all had metastasized disease, stage IIIb-IV, with previous treatments at the start of treatment with ipilimumab or the TIL treatment.

**Table 1:** Input parameters of base case, including survival probabilities, utilities and costs

Parameters	Mean	SE	Distribution	Source
Survival probabilities per year				
Ipilimumab				
PFS	0.175	0.012	Beta	[6]
OS	0.366	0.018	Beta	[6]
TIL				
PFS	0.234	0.089^a^	Beta	[10, 11]
OS	0.412	0.098^a^	Beta	[10, 11]
Utilities and side effects				
Ipilimumab				
Stable disease	0.850	0.020	Beta	[18]
Progression	0.590	0.020	Beta	[18]
TIL				
Stable disease	0.850	0.020	Beta	[18]
Progression	0.590	0.020	Beta	[18]
Utility decrements				
Fatigue	0.090	0.020	Beta	[18]
Diarrhea	0.060	0.020	Beta	[18]
Colitis	0.130	0.020	Beta	[18]
Neutropenia	0.130	0.020	Beta	[18]
Dyspnea	0.100	0.020	Beta	[18]
Flu-like syndrome (grade I/II))	0.090	0.020	Beta	[18]
Anaemia	0.110	0.020	Beta	[18]
Likelihood of side effects				
Ipilimumab				
Fatigue	0.070	0.015	Beta	[6]
Diarrhea	0.060	0.015	Beta	[6]
Colitis	0.060	0.015	Beta	[6]
Dyspnea	0.040	0.015	Beta	[6]
Immune	0.100	0.015	Beta	[6]
Anaemia	0.030	0.015	Beta	[6]
TIL^a^				
Fatigue	0.001	0.001	Beta	[24]
Diarrhea	0.001	0.001	Beta	[24]
Neutropenia	0.560	0.100	Beta	[24]
Dyspnea	0.020	0.015	Beta	[24]
Immune	0.220	0.100	Beta	[24]
Anaemia	0.440	0.100	Beta	[24]
Failures, non-compliance TIL				
Failures	0.100	0.015	Beta	[20], Expert opinion
Non-compliance	0.100	0.015	Beta	[21]
Costs in euros				
Costs of ipilimumab total	91,487.50	+/-25%	Gamma	
Drug	90,100.00	+/-25%	Gamma	[22]
Administration	473.00	+/-25%	Gamma	[23]
Management of side effects	914.50	+/-25%	Gamma	[6, 16]
Costs of TIL total	62,000.00	+/-25%	Gamma	NKI-AVL
TIL-production-total^b^	35,500.00	+/-25%	Gamma	NKI-AVL
Personnel	18,000.00	+/-25%	Gamma	NKI-AVL
Material and quality control	10,000.00	+/-25%	Gamma	NKI-AVL
Cleanroom and equipment	7,500.00	+/-25%	Gamma	NKI-AVL
TIL-hospital-total	26,500.00	+/-25%	Gamma	NKI-AVL
admission	13,000.00	+/-25%	Gamma	NKI-AVL
Preparatory surgery	6,500.00	+/-25%	Gamma	NKI-AVL
Side-effects, medication, monitoring	6,500.00	+/-25%	Gamma	NKI-AVL
Costs of follow-up stable disease^c^	516.00	+/-25%	Gamma	[25]
Costs progressive disease^d^	9,125.00	+/-25%	Gamma	[31]
Costs of side effects for ipilimumab				
Fatigue	198.00	+/-25%	Gamma	[16]
Diarrhea	580.00	+/-25%	Gamma	[16]
Colitis/neutropenia^e^	1115.00	+/-25%	Gamma	[16]
Dyspnea	100.00	+/-25%	Gamma	Assumption
Immune	7,680.00	+/-25%	Gamma	[16]
Anaemia	898.00	+/-25%	Gamma	[16]

^a^Modeled in the first cycle of “stable disease”

^b^based on 10 TIL productions per year

^c^based on 4^a^ follow-up visit physician+CT scan (stable)

^d^cost for palliative care or end-stage disease care was based on the per diem cost of a palliative care unit

^e^resembles 2-5 days hospitalization for severe toxicity (grade III-IV)

PFS: Progression Free Survival, OS: Overall Survival; SE: Standard Error; NKI-AVL: Netherlands Cancer Institute-Antoni van Leeuwenhoek hospital

Input cost price calculation NKI-AVL: based on *N*= 10 patients from the pilot study

Inclusion criteria of the pilot study were: a resectable metastasis of at least 2-3cm; a sufficient heart, lung and kidney function; a maximum of 2 asymptomatic brain metastasis smaller than 1cm; not concurrently being treated with immune function-suppressing medication; not having auto-immune disorders; and a minimum expected life span of 3 months

### Health effects

Health-related quality of life was modeled by assigning utilities to the different health states, in which the utilities are finally expressed in quality adjusted life years (QALYs). The utility is the instantaneous value of a given health state, a QALY is a measure of health gain. In the QALY, both the quality and the quantity of life lived are included, where the utility (between 0 and 1) is multiplied with the additional life years lived. The utilities attached to the health states were adapted from Beusterien et al. [18]. Beusterien and colleagues used standard gamble to derive utilities elicited from 140 respondents in the United Kingdom and Australia for 13 health states. The utility value for “baseline disease” was 0.80, for “progressive disease” 0.52, and for “death” the utility value was 0. Because no data was available for TIL, we assumed the utilities were equal to ipilimumab. For adverse events and side effects, disutilities were subtracted, these data were available for both groups, see Table 1.

### Costs

The academic costs for TIL-treatment were measured at the Netherlands Cancer Institute for the ten patients (*n* = 10) that were treated with TIL in the pilot phase before the CED program. Costs included production of TILs, treatment, hospitalisation and management of direct side effects. The costs of adverse events were calculated in the price of the TIL treatment based on the ten pilot patients and the estimated percentages of occurrence. A total preliminary estimate cost for treating one patient with TIL of €62,000 was measured, see Table 1 for details. This academic price was also reported to the Dutch National Health Care Institute [19]. In a further publication, more details will be presented on the costs of TIL.

Around 20% of patients could not be treated with TIL, due to an estimated 10% lab failures [20] in producing TILs and 10% for patients who developed progressive disease between signing informed consent and re-infusion of TILs [21]. In the model, it was assumed that these patients are therefore treated with the remaining option ipilimumab, with the associated overall survival and costs. In case of TIL production failure, the costs for only TIL production were charged.

Costs of ipilimumab were based on Dutch official medication prices (https://www.medicijnkosten.nl/) [22], calculated based on one adult (average 70 kg) needing 5 vials, which cost €4,505 each. Costs for administering the drug were assumed to be around €473 [23]. The average drug costs for treating one patient were €90,100. The costs for treating side-effects were €914. The total costs for ipilimumab amounted therefore €91,487.

### Adverse events

The adverse events associated with TIL differ from those of ipilimumab. The adverse events of TIL are mostly caused by either the non-myeloablative chemotherapy or the interleukin-2 (IL2), and occur during or directly after the treatment. Thus, the costs of adverse events for TIL were already incorporated in the price of TIL, because they all appeared in the time of hospitalization for the TIL treatment. The probabilities of adverse events were obtained from the literature [24]. As the probabilities for adverse events were more detailed in the publication by Ellebaek [24], we could better link them to the adverse events of ipilimumab as reported in Hodi et al. [6]. For ipilimumab, adverse events were obtained from Hodi et al. [6], and the associated costs and disutilities were processed according to the National Institute of Clinical Excellence (NICE) appraisal from the UK [16], see Table 1.

### Model analysis

We programmed the model in Microsoft Excel, 2010 (Microsoft, Redmond, WA). Discounting was incorporated by a rate of 4% for future costs and 1.5% for future effects to their present value per year, as the Dutch guidelines prescribe [25]. The Incremental cost-effectiveness ratio (ICER) was calculated by dividing the difference in costs by the difference in Life Years (incremental (iLYs)) and difference in Quality Adjusted Life Years (incremental (iQALYs)). Uncertainty in the input parameters was handled probabilistically, by assigning distributions to parameters (Table 1) [26]. Parameter values were drawn at random from the assigned distributions, using Monte Carlo simulation with 10,000 iterations. The results of the simulation of the hypothetical cohort of 1000 patients are illustrated in a cost-effectiveness (CE) plane. Each quadrant indicates whether a strategy is more or less expensive and more or less effective [27]. Decision uncertainty was shown by cost-effectiveness acceptability curves (CEACs), which present the probability of cost-effectiveness of the two alternatives for a range of threshold values. Whether a strategy is deemed efficient depends on how much society is willing to pay for a gain in effect, which is referred to as the ceiling ratio [27]. In the Netherlands an informal ceiling ratio of €80,000 per QALY exists for diseases with a high symptom burden [Dutch Council for Health and Society].

### Sensitivity analyses

One-way sensitivity analyses were conducted to evaluate the influence of parameters that are surrounded by uncertainty on the results. All parameters were varied by +/− 25%, to identify those most influential. Separately, we calculated the scenario in case that the TIL production costs would increase, as well as the maximum price that TIL is allowed to have to remain cost-effective compared to ipilimumab. To generalize the results, we changed the discounting with 3,5% for both costs and outcomes.

### Value of information analysis (VOI)

In early stages of technology development and research, Value of information analysis (VOI) is used to support decisions on the focus and design of further research, as e.g. in the context of the CED program. In the analysis, the current uncertainty of the parameters, as well as their influence on the results, are taken into account [28]. The difference between the expected net benefit obtained using perfect information and the expected net benefit obtained in the presence of uncertainty is known as the expected value of perfect information (EVPI). Generally, information is valuable when there is great uncertainty surrounding a decision and when that decision likely affects a large number of people in a meaningful way. The EVPI can be interpreted as the maximum amount the decision maker would be willing to spend to obtain perfect information [29]. As beneficial population was used 400 advanced melanoma patients in the Netherlands per year. The incidence of advanced melanoma in the Netherlands is around 800 patients. The estimation is that around 50% of these patients will be eligible for TIL treatment (see inclusion criteria).

## Results

### Base case results

In our model, the mean total life years for ipilimumab were 0.58 LY (=7 months) and 0.38 QALYs. For TIL, this was 0.70 LY (=8.4 months) and 0.45 QALYs. The total costs for ipilimumab amounted to €94,705 and for TIL to €81,140. The deterministic ICER therefor resulted in a dominant situation: TIL dominated ipilimumab.

### Sensitivity analysis

**Fig. 2:**
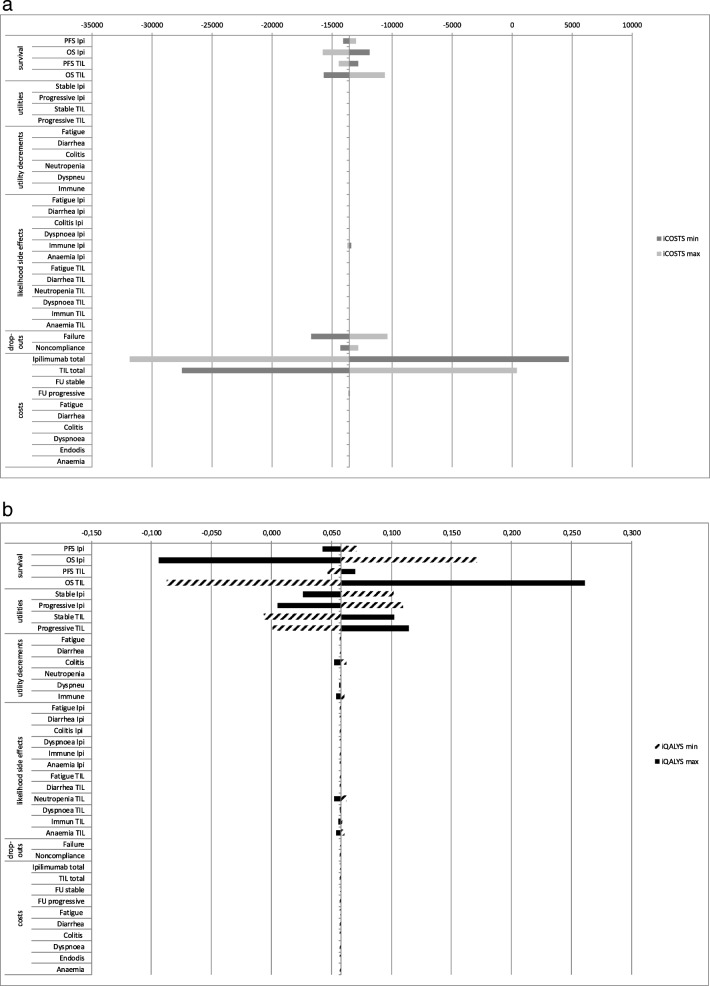
**a** Tornado diagram, presenting incremental costs results of sensitivity analyses. **b** Tornado diagram, presenting incremental qalys results of sensitivity analyses

The model outcomes proved robust against changes in model inputs. The parameters with the most impact on the incremental cost outcomes were survival, drop-outs and costs of treatment. For the incremental QALYs, these were survival and utilities, Fig. 2 and 2b. Yet, it would not change the result that TIL is expected to dominate ipilimumab. In the scenario that the production costs of TIL would increase up to €50,500 (assuming hospital costs remain equal), TIL would remain less costly, and still be more effective than ipilimumab at costs of up to €77,000 in total. Both the commonly used discount rates for the Netherlands (1,5% and 4%) as well of more commonly used percentages (3% and 3.5%) were used in the sensitivity analysis; this did not lead to additional findings. A sensitivity analysis for 3,5% discounting for both costs and outcomes resulted in somewhat lower QALYs; 0.43 QALYs and 0.37 QALYs for TIL and ipilimumab, and total costs of €81,172 for TIL and €94,732 for ipilimumab.

### Probabilistic sensitivity analysis

**Fig. 3:**
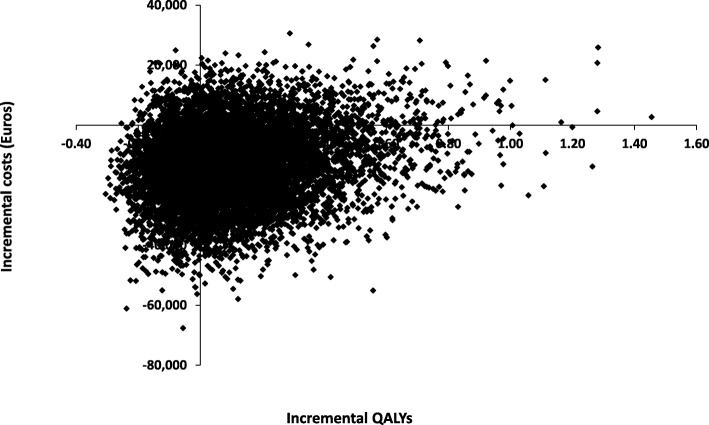
Cost-Effectiveness plane of the quality adjusted life years (QALYs) per costs of the TIL treatment versus ipilimumab. The scatter plot is showing the mean differences in costs and outcomes from the data using 10,000 bootstrap replicates. Fifty-six percent of the dots are in the South-East quadrant which indicates that the TIL treatment is in most cases less expensive and more effective compared to ipilimumab

**Fig. 4:**
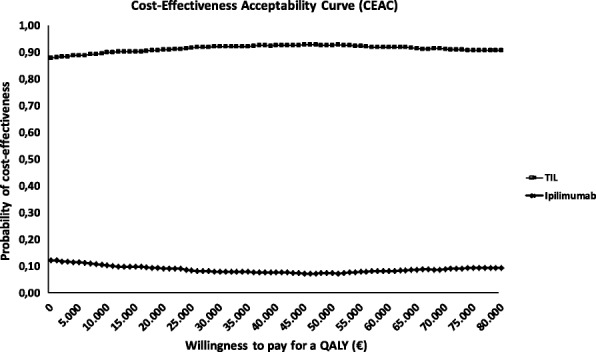
Base case Cost-effectiveness Acceptability Curves (CEAC); presenting the probability of cost-effectiveness of the two alternatives for a range of values of thresholds. Because the TIL is clearly dominant over ipilimumab, the lines do not cross each other.

The results of the probabilistic analysis showed that the TIL is more effective and less costly compared to ipilimumab. (See Fig. 3). The cost-effectiveness plane illustrates the costs and outcomes from 5,000 Monte Carlo simulations. In this scatterplot, 56% of the results were in the south-east (SE) quadrant, in which the new treatment is more effective and less costly. The remaining 44% were spread over the other three quadrants, which indicated a considerable amount of uncertainty. Figure 4 shows that TIL had the highest probability of being cost-effective at 92% certainty at a willingness to pay of €30,000/QALY, remained constant with a 91% certainty at a willingness to pay of €80,000/QALY (the latter is commonly used in the Netherlands for this type of treatment).

### Value of information analysis

**Fig. 5:**
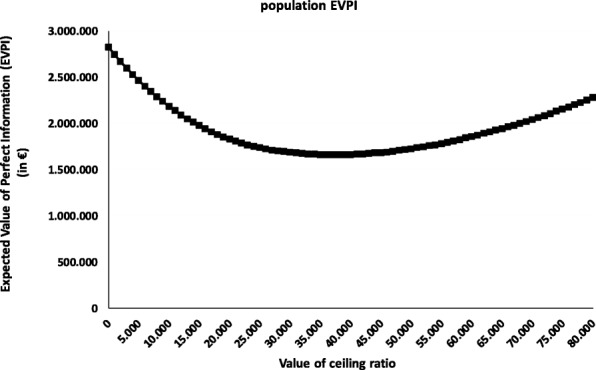
Expected Value of Perfect Information. The EVPI can be interpreted as the maximum amount the decision maker would be willing to spend to obtain perfect information. The Expected Value of Perfect Information (EVPI) amounted to almost €3 M, indicating the upper boundary for investing research funds in further clinical trials to obtain perfect information on the cost-effectiveness of TIL versus ipilimumab

Looking at the Cost-Effectiveness plane, where 56% of the dots are in the South-East quadrant (which indicates that the TIL treatment is in most cases less expensive and more effective compared to ipilimumab), the uncertainty in this stage is considerable (56% in the CE-plane, 86% in the CEAC). The Expected Value of Perfect Information (EVPI) amounted to almost €3 M, indicating the upper boundary for investing research funds in further clinical trials to obtain perfect information on the cost-effectiveness of TIL versus ipilimumab (Fig. 5).

## Discussion

As far as we know, this is the first cost-effectiveness analysis performed on an example of an Advanced Therapeutic Medicinal Products (ATMP): TIL treatment. Based on currently available results, TIL is expected to have the highest probability of being cost-effective compared to ipilimumab in advanced melanoma patients showing a higher effectiveness and lower costs. In the light of the new developments regarding nivolumab (whether or not in combination with ipilimumab), the lower costs are unique. This is a first, and thus early cost-effectiveness analysis, surrounded with substantial uncertainty. The second step is that the (cost-)effectiveness of TIL for advanced melanoma is currently compared in a randomized controlled trial as part of a CED program in the Netherlands. When this new data becomes available, the analysis will be updated, according to the iterative approach of Vallejo-Torres [28].

For our analysis, TIL versus ipilimumab, the ICER resulted in dominance for TIL (more effective, less costly). This means for the era of personalized medicine, cell therapy and ATMPs that more cost-effective options could be available to patients if these treatments are adopted and appropriately reimbursed in the health care system.

Limitation of our study are mostly due to the relatively early stage of TIL introduction and the various sources we used to gather the survival data for TIL, as there is not yet randomized controlled trial data available. Therefore, we pooled the survival data from two phase II studies, to account for the substantial amount of uncertainty in the existing evidence. As we are aware of the difference in level of evidence we compared in this analysis, we conducted sensitivity analyses which did account for possible variation. We further assumed that the utilities of patients treated with ipilimumab and TIL in the health states of the model were equal, since there is no data yet available for TIL. It is known that ipilimumab has more (long term) side effects, while the TIL treatment itself can have a short term-high impact on some patients. We based the current cost estimation of TIL on real world data obtained from 10 patients treated in the NKI, which is a small sample representing the final patient group. As part of the CED program, which was started in July 2015 in the Netherlands Cancer Institute, a randomized controlled trial including an Health Technology Assessment (HTA) will be conducted, in which survival, Health Related Quality of Life (HRQoL) and detailed cost data (direct and indirect) will be measured, in addition to survival.

At the other hand, the EVPI showed a relatively low amount (€3 M) necessary for further research. The TIL being part of a CED program, is an investment of the Dutch government of at least €6 M. This trial is necessary for the Dutch government to make a decision upon uptake of TIL in the basic coverage, to prove change “the state of science and practice”. However, maybe another (less costly) trial design could have been possible as well.

An important issue is, as recently was published by van Harten et al., that the price of several (expensive) drugs presently differs substantially between countries. This can influence the transferability of the results. The price for ipilimumab in Portugal was €2,975 per 50 mg, which means a total drug costs of €59,500, lower compared to TIL [30]. Obviously in such case the model outcomes will be different; probably resulting in TIL being “more costly, but more effective” compared to ipilimumab.

Furthermore, the costs of the TIL production were estimated based on the nonprofit setting of the NKI. Presently we can charge €62,000 per treatment, but meanwhile it has become clear that that amount is insufficient even on a non profit basis. If the TIL production would be taken over by a (pharmaceutical) company, it is very likely that the (commercial) price will increase considerably. This will decrease the price difference between TIL and ipilimumab, which might also change the results into “more costly, but more effective”. Our ICER calculations show that costs up to € 83,000 per treatment are still below the € 80,000/QALY threshold. This supports the proposals that were voiced to enter into innovative cooperation with industry or within science itself, to keep or refund the benefits of translational research to publicly funded agencies. Another important question is which patient subgroup will have the best response to TIL, which is currently unknown. The same is true for ipilimumab, nivolumab and/or pembrolizumab and even the combination of the first two in the future. If an appropriate biomarker would be found, that can identify which patients will respond to which treatment, this could again change the cost-effectiveness outcomes.

## Conclusion

Based on the current data, TIL is expected to have the highest probability of being cost-effective compared to ipilimumab in second line treatment for advanced melanoma. To further reduce decision uncertainty, a future clinical trial investigating costs and survival is most valuable, and this is currently undertaken as part of the CED program. During and after this trial, implementation support is necessary to overcome better accessibility.

## Abbreviations

95%-CI: 95% Confidence Interval
ATMP: Advanced Therapeutic Medicinal Products
CE plane: Cost Effectiveness Plane
CEA: Cost Effectiveness Analysis
CEAC: Cost Effectiveness Acceptability Curve
CED: Coverage with Evidence Development program
CHEERS: Consolidated Health Economic Evaluation Reporting Standards
DTIC: Dacarbazine
EVPI: Expected Value of Perfect Information
HRQoL: Health Related Quality of Health
HTA: Health Technology Assessment
ICER: Incremental Cost Effectiveness Ratio
iLY: Incremental Life Year
iQALY: Incremental Quality Adjusted Life Year
LY: Life Year
NKI: Netherlands Cancer Institute (Nederlands Kanker Instituut)
OS: Overall Survival
PFS: Progression Free Survival
PSA: Probabilistic Sensitivity Analysis
QALY: Quality Adjusted Life Year
RECIST: Response Evaluation Criteria In Solid Tumors
SE: Standard Error
TIL: Tumor Infiltrating Lymphocytes
VOI: Value of Information
